# Climate change accelerates winter transmission of a zoonotic pathogen

**DOI:** 10.1007/s13280-021-01594-y

**Published:** 2021-07-06

**Authors:** Saana Sipari, Hussein Khalil, Magnus Magnusson, Magnus Evander, Birger Hörnfeldt, Frauke Ecke

**Affiliations:** 1 Swedish University of Agricultural Sciences, Skogsmarksgränd, 901 83 Umeå, Sweden; 2 Umeå University, Department of Clinical Microbiology, 901 85 Umeå, Sweden

**Keywords:** Climate change, *Myodes glareolus*, North, Puumala orthohantavirus, Winter, Zoonosis

## Abstract

Many zoonotic diseases are weather sensitive, raising concern how their distribution and outbreaks will be affected by climate change. At northern high latitudes, the effect of global warming on especially winter conditions is strong. By using long term monitoring data (1980–1986 and 2003–2013) from Northern Europe on temperature, precipitation, an endemic zoonotic pathogen (Puumala orthohantavirus, PUUV) and its reservoir host (the bank vole, *Myodes glareolus*), we show that early winters have become increasingly wet, with a knock-on effect on pathogen transmission in its reservoir host population. Further, our study is the first to show a climate change effect on an endemic northern zoonosis, that is not induced by increased host abundance or distribution, demonstrating that climate change can also alter transmission intensity within host populations. Our results suggest that rainy early winters accelerate PUUV transmission in bank voles in winter, likely increasing the human zoonotic risk in the North.

**Supplementary Information:**

The online version contains supplementary material available at 10.1007/s13280-021-01594-y.

## Introduction

Global warming has been most pronounced in northern high latitude areas, i.e. Arctic tundra and boreal forest regions, where especially winters are getting warmer, wetter and more unstable. These changes and their consequences on northern ecosystems are raising concerns about the elevated risk of emergence of zoonotic diseases in these regions (Epstein 2001; Semenza and Menne 2009; Evengård and Sauerborn 2009; Randolph and Rogers 2010; Hueffer et al. 2013; Pauchard et al. 2016; Pecl et al. 2017; Contosta et al. 2019; Stoffel et al. 2020).

More than half of the known human pathogens are zoonotic (Jones et al. 2008), and the northward expanding distribution area of many zoonotic pathogens is a commonly projected effect of climate change on infectious diseases (Patz 1996; Semenza and Menne 2009; Evengård and Sauerborn 2009; Waits et al. 2018). However, the effect of climate change on endemic zoonoses that already exist in northern areas has received less attention (Parkinson and Evengård 2009; Evengård and Sauerborn 2009; Waits et al. 2018; Omazic et al. 2019). Many zoonoses found in the northern regions, such as Lyme borreliosis, tularemia, rabies, and orthohantavirus diseases are suggested to be weather sensitive (Lindgren et al. 2012; Omazic et al. 2019). Therefore, it is likely that local changes in the dynamics of endemic zoonoses will pose a more immediate risk on human health, compared to exotic, invasive pathogens. The need for improved knowledge on the mechanisms affecting and driving endemic zoonoses in the North is thus urgent.

Currently, several studies that suggest a relationship between weather and zoonotic disease transmission are based on models with pre-selected weather variables and human incidence data (Engelthaler et al. 1999; Pettersson et al. 2008; Clement et al. 2009; Xiao et al. 2013; Roda Gracia et al. 2015; Ma et al. 2019). While these studies are highly relevant in the field of epidemiology, they lack insight into the ecological mechanisms linking weather to human disease. Zoonotic diseases are transmitted via multilevel interactions between pathogens, reservoirs and/or vectors, humans and the environment. Thus, it is essential to understand the ecology of the interactions between pathogens and their reservoirs as well (Rohr et al. 2011; Altizer et al. 2013; Ostfeld and Brunner 2015; Cohen et al. 2020).

In our study, we used a unique, long-term data set to investigate the effect of climate change on the prevalence of a northern endemic pathogen, the Puumala orthohantavirus (PUUV, family *Hantaviridae*) in its only known reservoir host, the bank vole (*Myodes glareolus*). In boreal regions, bank vole populations are cyclic, and undergo notable density fluctuations with cycles of typically 3–5 years (Hansson and Henttonen 1985; Hornfeldt 1994; Cornulier et al. 2013). Earlier studies have shown a strong temporal effect of bank vole density on density of PUUV-infected voles, and consequently, increased risk of human infection (Niklasson et al. 1995; Kallio et al. 2009; Khalil et al. 2019). Humans are exposed to the virus through inhalation of aerosols contaminated with vole excreta. While chronic and mainly asymptomatic in the animal host (but see Kallio et al. 2007; Reil et al. 2017)), in humans PUUV causes nephropathia epidemica, a hemorrhagic fever with renal syndrome (Brummer-Korvenkontio et al. 1980; Vaheri et al. 2013).

Here, we tested the impact of changing winter conditions, driven by climate change, on PUUV prevalence in the reservoir host. As predictors for PUUV prevalence, we used bank vole densities and meteorological variables connected with climate change in the North, namely (1) amount of rain (mm) in autumn and winter months and (2) number of rainy days in autumn and winter months. The selected meteorological variables are in accordance with the main projected effects of climate change in the North, such as increased autumn and winter precipitation, delayed onset of winter, and decreased length of the snowy season (Rasmus et al. 2004; Jylhä et al. 2008; IPCC 2013). In addition, rainy winters are an important predictor of outbreaks of human nephropathia epidemica (Khalil et al. 2014), while the mechanism behind this relationship has remained unknown. Here, we introduce a missing piece to the puzzle: PUUV prevalence in the reservoir host. Our study fills an important knowledge gap on the processes driving PUUV outbreaks, enabling us to investigate the relationship between changing winter weather, pathogen-host interactions and human infection risk in northern high latitude regions. In our analyses, we used long term monitoring data on meteorological observations, and on bank vole population dynamics from Northern Sweden, from two different study periods; 1980–1986, and 2003–2013.

## Materials and methods

### Ethics statement

Trapping of animals was approved by the Swedish Environmental Protection Agency (latest permission: NV-01124-15) and the Animal Ethics Committee in Umeå (latest permissions: Dnr A 61-11 and A121-11), and all applicable institutional and national guidelines for the use of animals were followed.

### Study area and design

The study area was located in the eastern part of Västerbotten County, in the middle boreal subzone of northern Sweden (Sjörs 1999). The forest landscape is dominated by boreal coniferous forests and heavily influenced by forestry (Ecke et al. 2013). The 100 × 100 km study area comprises 16 regularly distributed 5 × 5 km sub-areas with an interdistance of 20 km (see (Hörnfeldt 1994) for detailed study design and map). In each 5 × 5 km sub-area, four 1-ha plots were systematically placed (unless a 1-ha plot hit water (*n* = 6)), yielding in total 58 plots. Within each 1-ha plot, a 90 m transect run diagonally with 10 trap-stations, each constituting five snap-traps. In each 1-ha plot small mammals were trapped for three consecutive nights, with dried apple and Polish wicks as a bait (Hörnfeldt 1994). The small mammal monitoring has been performed in spring and autumn, since autumn 1971 as part of the National Environmental Monitoring Programme (NEMP) (Ecke and Hörnfeldt 2021). All trapped specimens are biobanked at − 20 °C since autumn 1979.

### Bank vole and PUUV antibody data

The bank vole data used in this study comprised 7091 individuals, trapped as part of the NEMP from autumn 1980 to spring 1986 (2334 individuals) and autumn 2003 to autumn 2013 (4757 individuals) (Niklasson et al. 1995; Khalil et al. 2016, 2019). As an index of bank vole density we used the bank vole trapping index (number of trapped animals per 100 trap-nights); for simplicity here referred to as “density”. Median spring vole density, viz. 0.69, was used as a threshold value; spring trapping index values above 0.69 were here considered as a relatively high spring vole density.

Individuals weighing > = 14.4 g (5962 individuals) were screened for PUUV antibodies to assess the infection status (infected vs. uninfected) of the animals. We used enzyme-linked immunosorbent assay (ELISA) to detect anti-PUUV IgG antibodies in lung biopsies and to identify sero-positive individuals (Niklasson et al. 1995; Lindkvist et al. 2008; Khalil et al. 2016). Individuals weighing < 14.4 g were treated as juveniles and considered as still carrying maternal antibodies and thus not being infected (Kallio et al. 2006b). These animals were not included in any PUUV-analyses (Magnusson et al. 2015a; Khalil et al. 2016, 2019). Of the screened animals, 1349 animals (22.6%) were PUUV antibody seropositive and termed infected since PUUV causes a life-long persistent infection in bank voles (Meyer and Schmaljohn 2000).

### Meteorological data

All meteorological data used in this study was received from the Svartberget Research Station (Mellander et al. 2007) near Vindeln, Northern Sweden, located within the NEMP area and part of the Swedish Infrastructure for Ecosystem Science (SITES). The meteorological data covers diurnal average temperature and amount of precipitation (mm) for the periods of 1980–1986 and 2003–2013. Following Hansen et al. (2013) and Khalil et al. (2014), we calculated the number of rainy days and total amount of rain (mm) for late autumn (October), early winter (November), and for the mid-winter period (December to March) in each year. Days were classified as rainy if the following conditions were met: (1) average diurnal temperature was higher than 0 °C and (2) more than 1 mm precipitation was recorded. Precipitation was classified as snow, when average diurnal temperature was ≤ 0 °C. The selected seasons and meteorological variables are in accordance with the main projected effects of climate change in the North, such as increased autumn and winter precipitation, delayed onset of winter and decreased length of the snowy season (Rasmus et al. 2004; Jylhä et al. 2008; IPCC 2013).

### Statistical analyses

We used t-tests to compare 1) bank vole density, and 2) differences in mean temperatures, rainfall (mm), and number of rainy days for October, November and the mid-winter period (December-March), between the two study periods of 1980–1986 and 2003–2013. Linear regression was used to test the effect of November rain (mm, year *t*−1) on spring bank vole density (year *t*).

In addition to current spring (year *t*) and previous autumn densities (year *t*−1), we tested whether November rain (mm, year *t*−1), number of rainy days in November (year *t*−1), and mean November temperature (year *t*−1) explained the variation in PUUV seroprevalence in bank voles in current spring (year *t*). Additionally, we tested the relationship of rain (mm), number of rainy days, and mean temperature in preceding October (year *t*−1), and in mid-winter (December–March) with PUUV spring seroprevalence. Bank vole seroprevalence in spring was modeled as the number of positive individuals out of the total number of trapped voles in the whole study area using a generalized linear mixed effects model (glm) with binomial error distribution. The candidate explanatory variables included spring vole density (year *t*), preceding autumn vole density (year *t*−1), and weather variables (rain in mm, number of rainy days, mean temperature) for preceding October, November, and mid-winter (December–March) (See Supplementary Table S2).

For all models, we used Akaike Information Criteria (AIC) for model selection, and selected the model with the lowest AIC value. If two or more models were within 2(∆) AIC values, we selected the most parsimonious model. All analyses were performed in R using lme4 and tidyverse packages (Bates et al. 2015, Wickham et al. 2019).

## Results

**Fig. 1:**
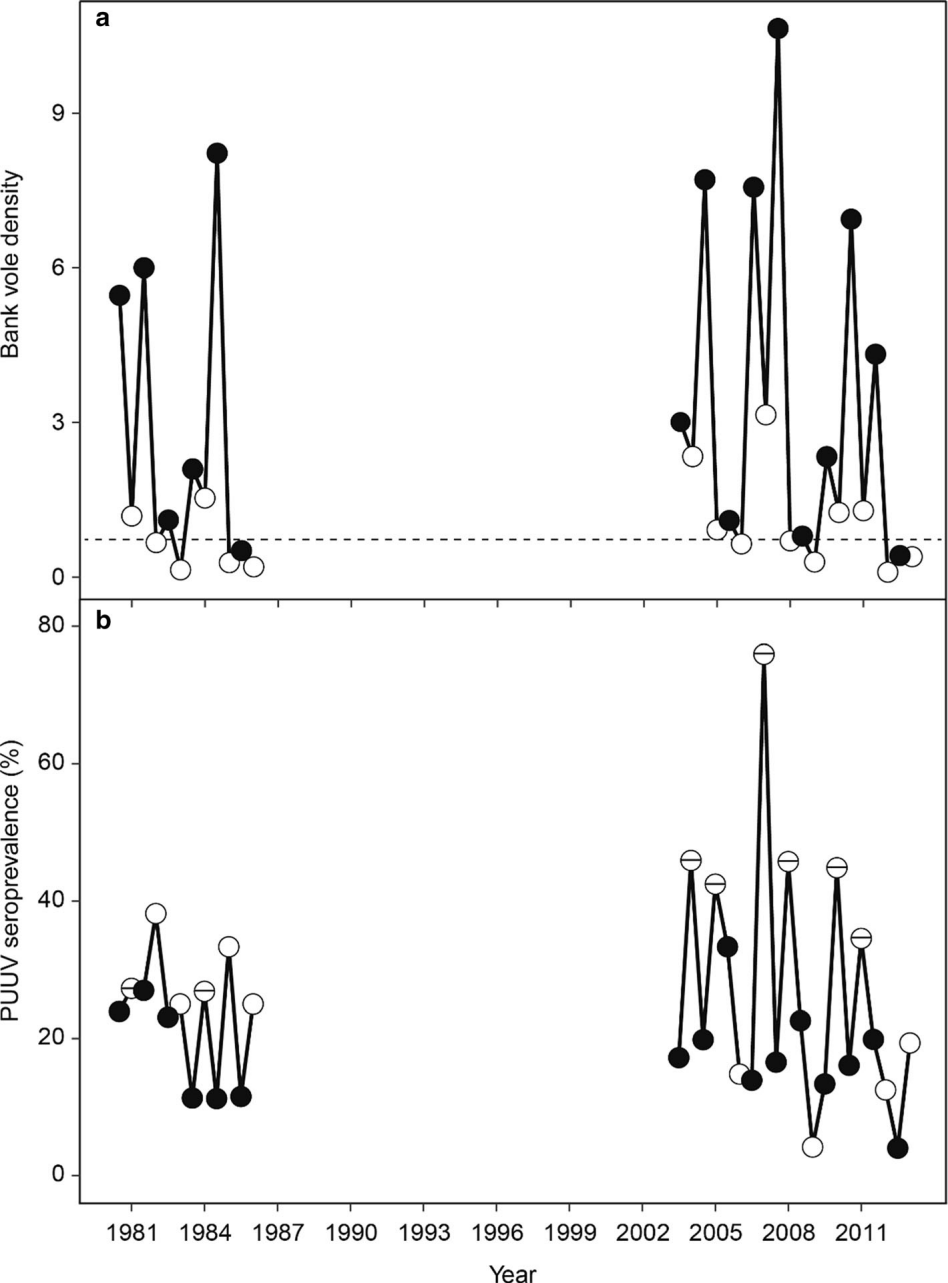
**a** Bank vole density (**a**) and PUUV seroprevalence in bank voles (**b**) in the study periods 1980–1986 and 2003–2013. While density did not differ between the 1980s and 2000s, in years with spring density above median (0.69, dashed reference line), PUUV seroprevalence in spring was significantly higher in the 2000s (*p* < 0.01). In **b** springs with bank vole density > 0.69 are indicated by white circles with a horizontal line. White and black circles represent spring and autumn trappings, respectively

**Fig. 2:**
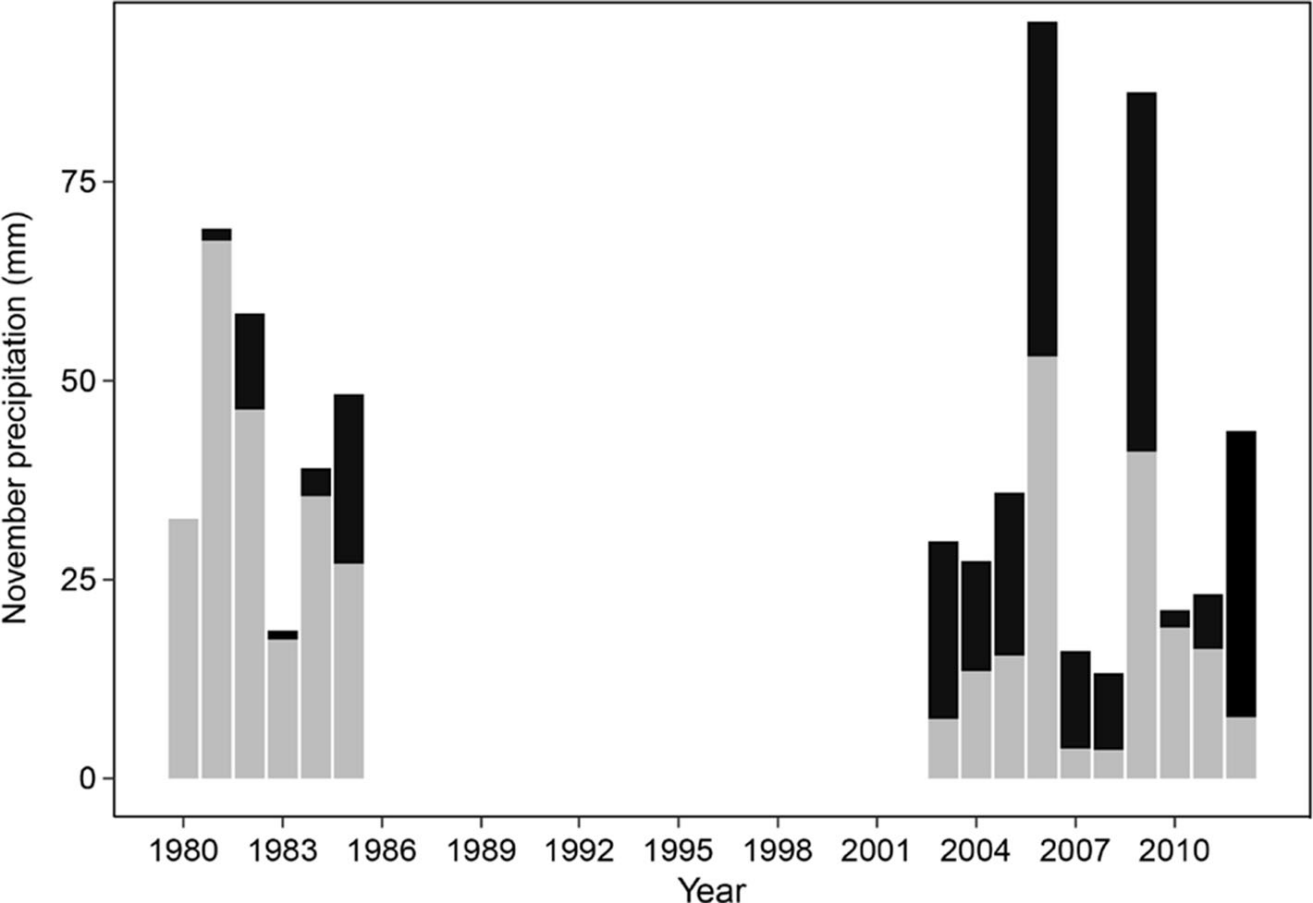
The amount of November precipitation (mm) during the study periods in the 1980s and 2000s (rain: black, snow: grey). The amount of rain in November was significantly higher in the 2000s (*p* < 0.05)

While bank vole density did not differ between the 2000s and 1980s study periods (Fig. 1a, Table 1), the PUUV spring seroprevalence in bank voles was significantly higher in the 2000s, but only during years above median spring density (trapping index > 0.69, see Material and methods, Fig. 1b, Table 1). At the same time, the average November temperature in the study area was notably higher during the 2000s study period, resulting in significantly wetter early winters (Fig. 2, Table 1). The amount of rain (mm) and the number of rainy days in November were both higher in the 2000s compared to the 1980s (Fig. 2, Table 1). In contrast, the number of rainy days and the total amount of rain in October, and mid-winter period (December-March, see Material and methods) did not differ between the 2000s and 1980s (Table 1).

**Table 1:** Mean values (± standard error, SE) and associated t-test results for bank vole autumn density, PUUV spring seroprevalence, and weather variables in the two study periods in the 1980s and 2000s. Significant differences (p < 0.05) are highlighted in bold. Winter represents December–March

Variables	1980s (mean ± SE)	2000s (mean ± SE)	*T*	*df*	*p*
Bank vole autumn density	3.9 ± 1.3	4.5 ± 1.1	− 0.344	11.83	0.737
Bank vole spring density	0.7 ± 0.2	1.1 ± 0.3	− 1.141	13.98	0.273
PUUV spring seroprevalence	28.6 ± 4.9	32.1 ± 5.9	− 0.566	11.24	0.583
PUUV spring seroprevalence with spring density > 0.69	26.4 ± 0.9	45.4 ± 3.3	− 5.501	5.55	**0.002**
November rain (mm)	86.6 ± 3.4	21.1 ± 4	− 2.463	13.98	**0.027**
Number of rainy days in November	1.7 ± 0.6	5.1 ± 1.2	− 2.634	12.97	**0.021**
November temperature (°C)	− 6.3 ± 1.0	− 2.8 ± 0.9	− 2.580	11.73	**0.024**
October rain (mm)	72.2 ± 12.4	41.4 ± 9.3	1.972	10.34	0.076
Number of rainy days in October	9.2 ± 1.7	7.1 ± 1.4	0.935	10.76	0.370
October temperature (°C)	1.7 ± 0.5	2.0 ± 0.4	− 0.361	11.66	0.725
Winter rain (mm)	8.4 ± 2.8	13.3 ± 5.0	− 0.851	13.15	0.410
Number of rainy days in winter	1.5 ± 0.4	3.1 ± 1.2	− 1.268	11.15	0.231
Winter temperature (°C)	− 9.6 ± 0.6	− 7.2 ± 0.6	− 2.965	12.83	0.011

**Fig. 3:**
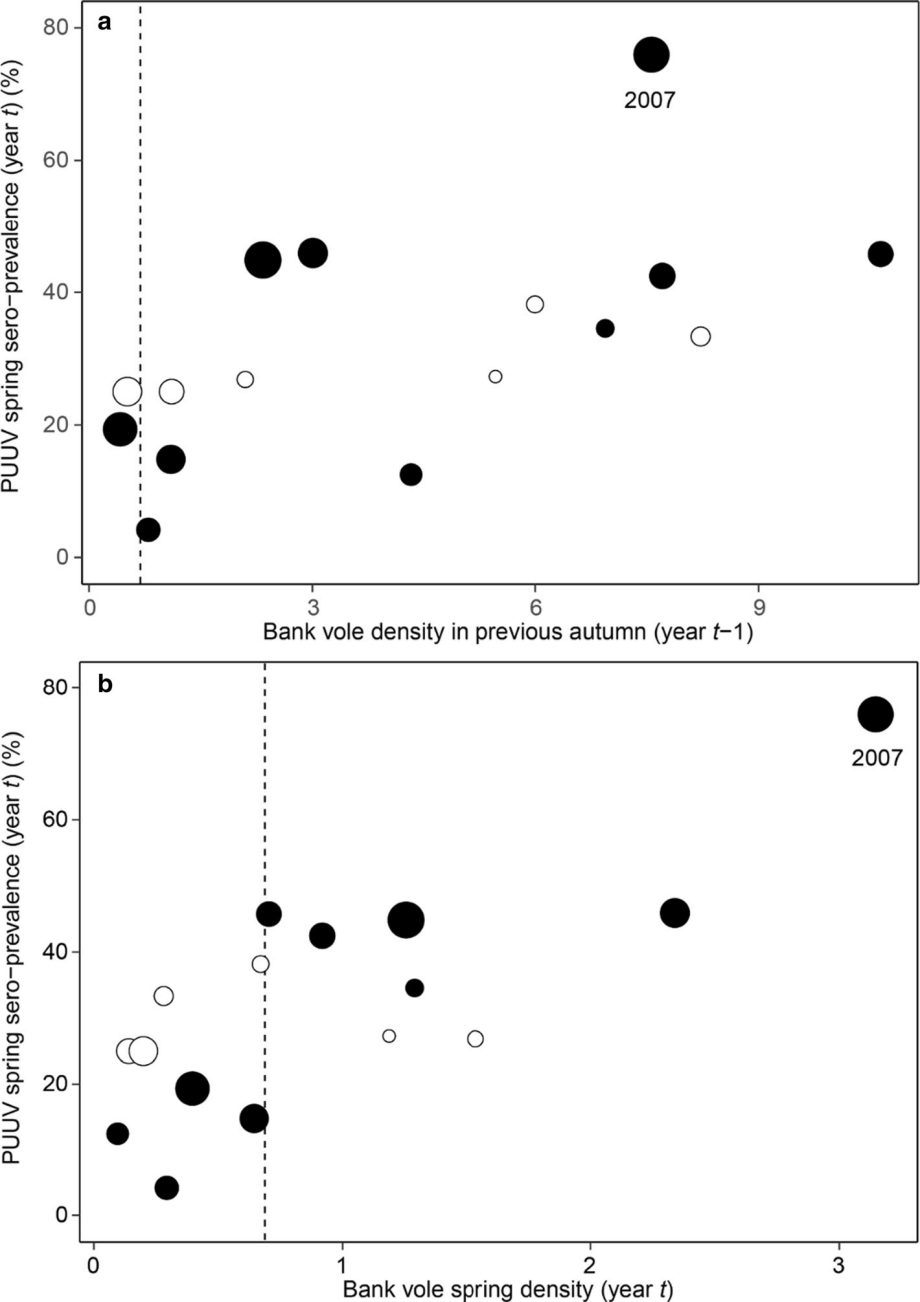
Relationship between PUUV spring seroprevalence in bank voles and their density in preceding autumn (**a**) and current spring (**b**); open circles represent 1980–1986 and closed circles 2003–2013. The amount of November rain (in mm, year *t*−1) is represented by different sized circles for 0–10, > 10–20, > 20–30, > 30–40, > 40 mm rain. The vertical dashed reference line represents median spring density (0.69). The outbreak of nephropathia epidemica in 2007 is labelled in the figure

Both previous autumn and current spring population densities, separately and combined, explained PUUV seroprevalence in spring in bank voles, and including November rain (mm) resulted in the most parsimonious model (Table 2, Fig. 3a, b). The number of rainy days in November also acted as a predictor of PUUV seroprevalence in spring, but the effect was weaker (see Supplementary Table S1). November rain did not affect bank vole density in spring (*t* = 1.518, *p* value = 0.151).

**Table 2:** The generalized linear model with the variables best predicting PUUV seroprevalence in spring in the bank vole population (*n* = 16 years)

Predictors	Odds ratios	CI	*p*
Intercept	0.12	0.09–0.17	< 0.001
Bank vole autumn density (year *t*−1)	1.15	1.10–1.20	< 0.001
Bank vole autumn density (year *t*)	1.58	1.34–1.87	< 0.001
November rain (in mm, year *t*−1)	1.02	1.01–1.03	< 0.001

## Discussion

To our knowledge, our study is the first one to demonstrate a climate change effect on an endemic northern zoonosis that is not induced by increased host density or expansion of the host’s geographical distribution. Our results suggest that the increasingly wetter early winters in the North are associated with higher PUUV spring seroprevalence in the reservoir host, the bank vole, and very likely, with increased risk of human infection.

Our study provides new insights into the role of environmental factors in orthohantavirus transmission dynamics at a larger scale. So far, the relationship between precipitation and orthohantavirus prevalence in reservoir species or humans in other parts of the world (Asia, Americas and Central Europe) has been attributed to reservoir population growth following heavy rains and increased plant production (Engelthaler et al. 1999; Yates et al. 2002; Clement et al. 2009; Donalisio and Peterson 2011; Xiao et al. 2013; Tian et al. 2017). In our study, host density was not affected, suggesting that climate-induced mechanisms in pathogen transmission are diverse and still poorly understood.

At northern high latitudes, small rodents are vectors and reservoirs for many endemic zoonoses, such as Lyme borreliosis, tularemia and orthohantavirus diseases (Kruse et al. 2004). As temperature increases (IPCC 2013), the resulting warmer, wetter and increasingly unstable winters are expected to play an important role in the overwintering success of the rodents (Aars and Ims 2002; Hörnfeldt 2004; Kausrud et al. 2008; Cornulier et al. 2013; Magnusson et al. 2015b), with a potential effect on pathogen transmission in the population. Our results suggest that a limited period of time in the beginning of the winter (November), instead of weather conditions during the whole winter, can play an important role in the dynamics of PUUV transmission and the resulting spring prevalence in bank voles. The effect of November rain on PUUV seroprevalence in bank voles as seen in our model (Table 2) is seemingly small, yet, it is ecologically relevant. If the winter warming in the North continues to escalate as projected (IPCC 2013), the impact of wet early winters on PUUV transmission is likely to amplify.

**Fig. 4:**
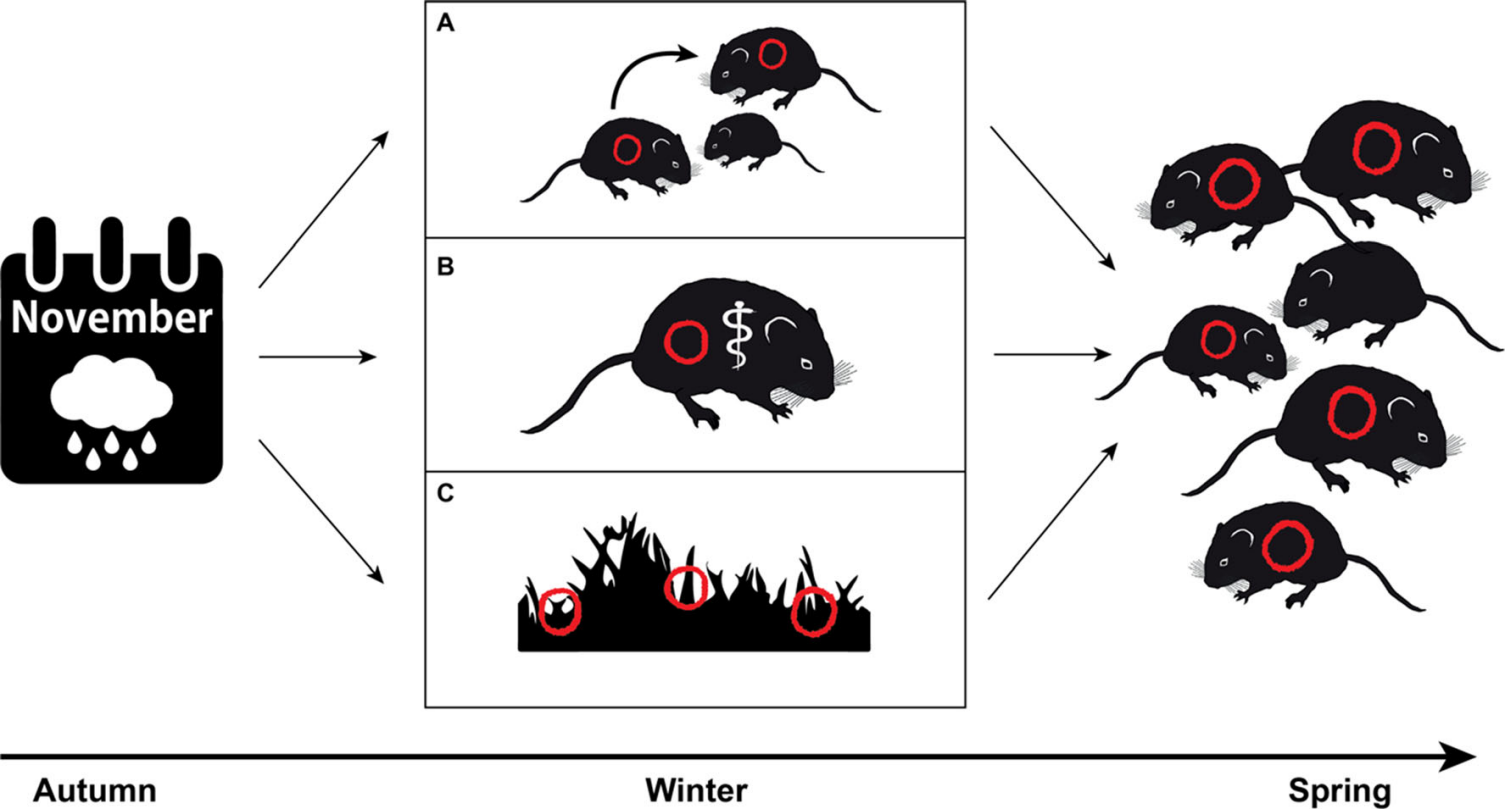
Illustration of three mutually non-exclusive mechanistic processes (A–C) that may explain why high amounts of rain instead of snow in November contribute to high spring seroprevalence of the Puumala orthohantavirus (red symbols). Adverse environmental conditions, incl. heavy rains, and recurrent thaws and freezing periods, with ice bark formation and filling of holes and cavities with water and ice (A) force bank voles to increase aggregating in winter and increase their social contact in remaining and less abundant micro-habitats, resulting in increased transmission of the virus, (B) cause environmental stress in bank voles and impair their physiological condition (symbolized by rod of Asclepius) making them more susceptible for the virus and/or (C) increase the environmental persistence of the virus compared to Novembers characterized by less humid conditions and hence increase the risk of environmental transmission of the virus to the bank voles

Here, we describe three mutually non-exclusive, testable mechanisms that could explain how wet early winters may enhance PUUV transmission in bank voles: (A) altered host behavior, (B) impaired host physiology, and (C) increased environmental persistence of the pathogen (Fig. 4).

In light of our findings, we state that climate change has already increased the risk of zoonotic infections and human health in the North. In the year 2007, an unprecedented outbreak of nephropathia epidemica hit northern Sweden, and acts as a striking example (Pettersson et al. 2008; Evander and Ahlm 2009; Olsson et al. 2009). Our results show, how the high vole population density in the autumn 2006 was followed by an exceptionally rainy November (Figs. 2 and 3). Consequently, spring 2007 PUUV seroprevalence in voles turned out to be substantially higher than other years (Figs. 1 and 3). Further, in 2007, many people living in the area reported unusually high level of bank vole infestation inside houses (Pettersson et al. 2008). It seems that the high host density in autumn 2006 combined with a rainy onset of winter promoted the pathogen transmission in the vole population. The wet winter likely drove voles into human dwellings to seek shelter, exposing humans to a high number of infected animals, resulting in an epidemic with historically high number of human cases in the area (Pettersson et al. 2008; Evander and Ahlm 2009; Olsson et al. 2009; Khalil et al. 2014).

In addition to PUUV, there are several other endemic northern zoonotic pathogens, potentially sensitive for the changing climate conditions, with a marked effect on human health (Lindgren et al. 2012). For instance, the effects of climate change on tick-borne-pathogens have been increasingly recognized. *Ixodes* spp. ticks have expanded their latitudinal and altitudinal distribution, and cases of tick-transmitted diseases, such as Lyme borreliosis and tick-borne-encephalitis (TBE), have increased over time (Jore et al. 2011; Medlock et al. 2013; Ostfeld and Brunner 2015; Kjær et al. 2019)^.^ Tularemia, a disease caused by the bacterium *Francisella tularensis,* involving small mammals, blood feeding insects and acari in its complicated life cycle, has also increased during the last decades (Larssen et al. 2014; Desvars et al. 2015; Ma et al. 2019). In North America and Siberia, both rabies and a lethal helminthic disease caused by *Echinococcus multilocularis* are expected to benefit from warmer winters (Parkinson and Butler 2005; Hueffer et al. 2013).

Global warming causing increasingly shorter and warmer winters at northern latitudes offers a plausible explanation for some of the already observed changes in the dynamics of zoonotic diseases, yet, the actual underlying mechanisms connecting the recent trends with climate change are poorly understood. Supported by our study, introducing reservoir dynamics in epidemiological models of zoonotic diseases is important, and will improve the accuracy and reliability of such models. Further, the climate-induced mechanisms in pathogen transmission can be diverse, and likely vary with latitude and altitude. Our results suggest that even seemingly modest changes in climate, such as increased rainfall during one particular winter month, can have a significant effect on pathogen transmission, and thus, potentially on human health (Lindgren et al. 2012). It remains unclear if the here identified climate variables are also of relevance for other zoonoses in the North. PUUV shows environmental persistence and this persistence is likely benefitting from increased November rain (Kallio et al. 2006a) (mechanism C, Fig. 4). It is therefore important to also study potential climate responses in other zoonoses caused by pathogens with environmental persistence such as tularemia (Schulze et al. 2016), and to examine if climate change increases pathogen prevalence in the reservoir hosts and/or incidence in humans.

More detailed knowledge on the ecology of reservoir species during winter and their interaction with pathogens, and also survival of the pathogens themselves during warmer and wetter winters, is necessary to gain a mechanistic understanding of the effect of climate warming on emergence and dynamics of zoonotic diseases in the North. For PUUV and other horizontally transmitted pathogens occurring in snow covered areas on the northern and southern hemisphere, testing the three suggested mechanisms and their respective effect on disease transmission described in Fig. 4 should be pivotal.

## Supplementary Information

Below is the link to the electronic supplementary material.

Top five models (lowest AICc values) best predicting PUUV spring seroprevalence in bank voles. All candidate variables included in comparison of all model subsets are listed below the table. Supplementary file1 (PDF 626 kb)

## References

[CR1] Aars J, Ims RA (2002). Intrinsic and climatic determinants of population demography: The winter dynamics of tundra voles. Ecology.

[CR2] Altizer S, Ostfeld RS, Johnson PTJ, Kutz S, Harvell CD (2013). Climate change and infectious diseases: From evidence to a predictive framework. Science.

[CR3] Bates D, Mächler M, Bolker B, Walker S (2015). Fitting linear mixed-effects models using lme4. Journal of Statistical Software.

[CR4] Brummer-Korvenkontio M, Vaheri A, Hovi T, von Bonsdorff CH, Vuorimies J, Manni T, Penttinen K, Oker-Blom N (1980). Nephropathia epidemica: Detection of antigen in bank voles and serologic diagnosis of human infection. Journal of Infectious Diseases.

[CR5] Clement J, Vercauteren J, Verstraeten WW, Ducoffre G, Barrios JM, Vandamme AM, Maes P, Van Ranst M (2009). Relating increasing hantavirus incidences to the changing climate: The mast connection. International Journal of Health Geographics.

[CR6] Cohen JM, Sauer EL, Santiago O, Spencer S, Rohr JR (2020). Divergent impacts of warming weather on wildlife disease risk across climates. Science.

[CR7] Contosta AR, Casson NJ, Garlick S, Nelson SJ, Ayres MP, Burakowski EA, Campbell J, Creed I (2019). Northern forest winters have lost cold, snowy conditions that are important for ecosystems and human communities. Ecological Applications.

[CR8] Cornulier T, Yoccoz NG, Bretagnolle V, Brommer JE, Butet A, Ecke F, Elston DA, Framstad E (2013). Europe-wide dampening of population cycles in keystone herbivores. Science.

[CR9] Desvars A, Furberg M, Hjertqvist M, Vidman L, Sjöstedt A, Rydén P, Johansson A (2015). Epidemiology and ecology of Tularemia in Sweden, 1984–2012. Emerging Infectious Diseases.

[CR10] Donalisio MR, Peterson AT (2011). Environmental factors affecting transmission risk for hantaviruses in forested portions of southern Brazil. Acta Tropica.

[CR11] Ecke, F., and B. Hörnfeldt. 2021. Miljöövervakning av smågnagare. http://www.slu.se/mo-smagnagare.

[CR12] Ecke F, Magnusson M, Hörnfeldt B (2013). Spatiotemporal changes in the landscape structure of forests in northern Sweden. Scandinavian Journal of Forest Research.

[CR13] Engelthaler DM, Mosley DG, Cheek JE, Levy CE, Komatsu KK, Ettestad P, Davis T, Tanda DT (1999). Climatic and environmental patterns associated with hantavirus pulmonary syndrome, four corners region, United States. Emerging Infectious Diseases.

[CR14] Epstein PR (2001). Climate change and emerging infectious diseases. Microbes and Infection.

[CR15] Evander M, Ahlm C (2009). Milder winters in northern Scandinavia may contribute to larger outbreaks of haemorrhagic fever virus. Global Health Action.

[CR16] Evengård B, Sauerborn R (2009). Climate change influences infectious diseases both in the Arctic and the tropics: Joining the dots. Global Health Action.

[CR17] Hansen BB, Grøtan V, Aanes R, Sæther BE, Stien A, Fuglei E, Ims RA, Yoccoz NG (2013). Climate events synchronize the dynamics of a resident vertebrate community in the high arctic. Science.

[CR18] Hansson L, Henttonen H (1985). Gradients in density variations of small rodents: The importance of latitude and snow cover. Oecologia.

[CR19] Hörnfeldt Birger (1994). Delayed density dependence as a determinant of vole cycles. Ecology.

[CR20] Hörnfeldt B (2004). Long-term decline in numbers of cyclic voles in boreal Sweden: Analysis and presentation of hypotheses. Oikos.

[CR21] Hueffer K, Parkinson AJ, Gerlach R, Berner J (2013). Zoonotic infections in Alaska: Disease prevalence, potential impact of climate change and recommended actions for earlier disease detection, research, prevention and control. International Journal of Circumpolar Health.

[CR62] IPCC, 2013: Climate Change 2013: The Physical Science Basis. Contribution of Working Group I to the Fifth Assessment Report of the Intergovernmental Panel on Climate Change [Stocker, T.F., D. Qin, G.-K. Plattner, M. Tignor, S.K. Allen, J. Boschung, A. Nauels, Y. Xia, V. Bex and P.M. Midgley (eds.)]. Cambridge University Press, Cambridge, United Kingdom and New York, NY, USA, 1535 pp.

[CR22] Jones KE, Patel NG, Levy MA, Storeygard A, Balk D, Gittleman JL, Daszak P (2008). Global trends in emerging infectious diseases. Nature.

[CR23] Jore S, Viljugrein H, Hofshagen M, Brun-Hansen H, Kristoffersen AB, Nygård K, Brun E, Ottesen P (2011). Multi-source analysis reveals latitudinal and altitudinal shifts in range of Ixodes ricinus at its northern distribution limit. Parasites and Vectors.

[CR24] Jylhä K, Fronzek S, Tuomenvirta H, Carter TR, Ruosteenoja K (2008). Changes in frost, snow and Baltic sea ice by the end of the twenty-first century based on climate model projections for Europe. Climatic Change.

[CR25] Kallio ER, Begon M, Henttonen H, Koskela E, Mappes T, Vaheri A, Vapalahti O (2009). Cyclic hantavirus epidemics in humans: Predicted by rodent host dynamics. Epidemics.

[CR26] Kallio ER, Klingström J, Gustafsson E, Manni T, Vaheri A, Henttonen H, Vapalahti O, Lundkvist Å (2006). Prolonged survival of Puumala hantavirus outside the host: Evidence for indirect transmission via the environment. Journal of General Virology.

[CR27] Kallio ER, Poikonen A, Vaheri A, Vapalahti O, Henttonen H, Koskela E, Mappes T (2006). Maternal antibodies postpone hantavirus infection and enhance individual breeding success. Proceedings of the Royal Society B.

[CR28] Kallio ER, Voutilainen L, Vapalahti O, Vaheri A, Henttonen H, Koskela E, Mappes T (2007). Endemic hantavirus infection impairs the winter survival of its rodent host. Ecology.

[CR29] Kausrud KL, Mysterud A, Steen H, Vik JO, Østbye E, Cazelles B, Framstad E, Eikeset AM (2008). Linking climate change to lemming cycles. Nature.

[CR30] Khalil H, Ecke F, Evander M, Bucht G, Hörnfeldt B (2019). Population dynamics of bank voles predicts human puumala hantavirus risk. EcoHealth.

[CR31] Khalil H, Ecke F, Evander M, Magnusson M, Hörnfeldt B (2016). Declining ecosystem health and the dilution effect. Scientific Reports.

[CR32] Khalil H, Olsson G, Ecke F, Evander M, Hjertqvist M, Magnusson M, Löfvenius MO, Hörnfeldt B (2014). The importance of bank vole density and rainy winters in predicting nephropathia epidemica incidence in northern Sweden. PLoS ONE.

[CR33] Kjær LJ, Soleng A, Edgar KS, Lindstedt HEH, Paulsen KM, Andreassen ÅK, Korslund L, Kjelland V (2019). Predicting and mapping human risk of exposure to Ixodes ricinus nymphs using climatic and environmental data, Denmark, 2016. Norway and Sweden. Eurosurveillance.

[CR34] Kruse H, Kirkemo AM, Handeland K (2004). Wildlife as source of zoonotic infections. Emerging Infectious Diseases.

[CR35] Larssen KW, Bergh K, Heier BT, Vold L, Afset JE (2014). All-time high tularaemia incidence in Norway in 2011: Report from the national surveillance. European Journal of Clinical Microbiology and Infectious Diseases.

[CR36] Lindgren E, Andersson Y, Suk JE, Sudre B, Semenza JC (2012). Public health: Monitoring EU emerging infectious disease risk due to climate change. Science.

[CR37] Lindkvist M, Näslund J, Ahlm C, Bucht G (2008). Cross-reactive and serospecific epitopes of nucleocapsid proteins of three hantaviruses: Prospects for new diagnostic tools. Virus Research.

[CR38] Ma Y, Bring A, Kalantari Z, Destouni G (2019). Potential for hydroclimatically driven shifts in infectious disease outbreaks: The case of Tularemia in high-latitude regions. International Journal of Environmental Research and Public Health.

[CR39] Magnusson M, Ecke F, Khalil H, Olsson G, Evander M, Niklasson B, Hörnfeldt B (2015). Spatial and temporal variation of hantavirus bank vole infection in managed forest landscapes. Ecosphere.

[CR40] Magnusson M, Hörnfeldt B, Ecke F (2015). Evidence for different drivers behind long-term decline and depression of density in cyclic voles. Population Ecology.

[CR41] Medlock JM, Hansford KM, Bormane A, Derdakova M, Estrada-Peña A, George JC, Golovljova I, Jaenson TGT (2013). Driving forces for changes in geographical distribution of *Ixodes ricinus* ticks in Europe. Parasites and Vectors.

[CR42] Mellander PE, Löfvenius MO, Laudon H (2007). Climate change impact on snow and soil temperature in boreal Scots pine stands. Climatic Change.

[CR43] Meyer BJ, Schmaljohn CS (2000). Persistent hantavirus infections: Characteristics and mechanisms. Trends in Microbiology.

[CR44] Niklasson B, Hörnfeldt B, Lundkvist A, Björsten S, Leduc J (1995). Temporal dynamics of Puumala virus antibody prevalence in voles and of nephropathia epidemica incidence in humans. American Journal of Tropical Medicine and Hygiene.

[CR45] Olsson GE, Hjertqvist M, Lundkvist Å, Hörnfeldt B (2009). Predicting high risk for human hantavirus infections, Sweden. Emerging Infectious Diseases.

[CR46] Omazic A, Bylund H, Boqvist S, Högberg A, Björkman C, Tryland M, Evengård B, Koch A (2019). Identifying climate-sensitive infectious diseases in animals and humans in Northern regions. Acta Veterinaria Scandinavica.

[CR47] Ostfeld RS, Brunner JL (2015). Climate change and Ixodes tick-borne diseases of humans. Philosophical Transactions of the Royal Society B.

[CR48] Parkinson AJ, Butler JC (2005). Potential impacts of climate change on infectious diseases in the Arctic. International Journal of Circumpolar Health.

[CR49] Parkinson AJ, Evengård B (2009). Climate change, its impact on human health in the Arctic and the public health response to threats of emerging infectious diseases. Global Health Action.

[CR50] Patz JA (1996). Global climate change and emerging infectious diseases. The Journal of the American Medical Association.

[CR51] Pauchard A, Milbau A, Albihn A, Alexander J, Burgess T, Daehler C, Englund G, Essl F (2016). Non-native and native organisms moving into high elevation and high latitude ecosystems in an era of climate change: New challenges for ecology and conservation. Biological Invasions.

[CR52] Pecl GT, Araújo MB, Bell JD, Blanchard J, Bonebrake TC, Chen IC, Clark TD, Colwell RK (2017). Biodiversity redistribution under climate change: Impacts on ecosystems and human well-being. Science.

[CR53] Pettersson L, Boman J, Juto P, Evander M, Ahlm C (2008). Outbreak of Puumala virus infection, Sweden. Emerging Infectious Diseases.

[CR54] Randolph SE, Rogers DJ (2010). The arrival, establishment and spread of exotic diseases: Patterns and predictions. Nature Reviews Microbiology.

[CR55] Rasmus S, Räisänen J, Lehning M (2004). Estimating snow conditions in Finland in the late 21st century using the SNOWPACK model with regional climate scenario data as input. Annals of Glaciology.

[CR56] Reil D, Rosenfeld UM, Imholt C, Schmidt S, Ulrich RG, Eccard JA, Jacob J (2017). Puumala hantavirus infections in bank vole populations: Host and virus dynamics in Central Europe. BMC Ecology.

[CR57] Roda Gracia J, Schumann B, Seidler A (2015). Climate variability and the occurrence of human puumala hantavirus infections in Europe: A systematic review. Zoonoses and Public Health.

[CR58] Rohr JR, Dobson AP, Johnson PTJ, Kilpatrick AM, Paull SH, Raffel TR, Ruiz-Moreno D, Thomas MB (2011). Frontiers in climate change-disease research. Trends in Ecology and Evolution.

[CR59] Schulze C, Heuner K, Myrtennäs K, Karlsson E, Jacob D, Kutzer P, Große K, Forsman M (2016). High and novel genetic diversity of Francisella tularensis in Germany and indication of environmental persistence. Epidemiology and Infection.

[CR60] Semenza JC, Menne B (2009). Climate change and infectious diseases in Europe. The Lancet Infectious Diseases.

[CR61] Sjörs H (1999). The background: Geology, climate and zonation. Acta Phytogeographica Suecica.

[CR63] Stoffel M, Stephenson DB, Haywood JM (2020). Antipyretic medication for a feverish planet. Earth Systems and Environment.

[CR64] Tian H, Yu P, Cazelles B, Xu L, Tan H, Yang J, Huang S, Xu B (2017). Interannual cycles of Hantaan virus outbreaks at the human-animal interface in Central China are controlled by temperature and rainfall. Proceedings of the National Academy of Sciences.

[CR65] Vaheri A, Henttonen H, Voutilainen L, Mustonen J, Sironen T, Vapalahti O (2013). Hantavirus infections in Europe and their impact on public health. Reviews in Medical Virology.

[CR66] Waits A, Emelyanova A, Oksanen A, Abass K, Rautio A (2018). Human infectious diseases and the changing climate in the Arctic. Environment International.

[CR67] Wickham H, Averick M, Bryan J, Chang W, McGowan L, François R, Grolemund G, Hayes A (2019). Welcome to the Tidyverse. Journal of Open Source Software.

[CR68] Xiao H, Tian HY, Cazelles B, Li XJ, Tong SL, Gao LD, Qin JX, Lin XL (2013). Atmospheric moisture variability and transmission of hemorrhagic fever with renal syndrome in Changsha City, Mainland China, 1991–2010. PLoS Neglected Tropical Diseases.

[CR69] Yates TL, Mills JN, Parmenter CA, Ksiazek TG, Parmenter RR, Vande Castle JR, Calisher CH, Nichol ST (2002). The ecology and evolutionary history of an emergent disease: Hantavirus pulmonary syndrome. BioScience.

