# Supplementary material for: Climate change accelerates winter transmission of a zoonotic pathogen
Source: Ambio. 2021 Jul 6;51(3):508–17. doi: 10.1007/s13280-021-01594-y (PMC8800963; doi:10.1007/s13280-021-01594-y)
Supplement: Supplementary file 1 — Top five models (lowest AICc values) best predicting PUUV spring seroprevalence in bank voles. All candidate variables included in comparison of all model subsets are listed below the table. Supplementary file1 (PDF 626 kb) [file 13280_2021_1594_MOESM1_ESM.pdf]

Ambio

Electronic Supplementary Material
This supplementary material has not been peer reviewed.

Title: **Climate change accelerates winter transmission of a zoonotic pathogen**

Supplementary table 1. Top five models (lowest AICc values) best predicting PUUV spring seroprevalence bank voles. All candidate variables included in comparison of all model subsets are listed below the table.

Model Rank	Variables	Log likelihood	AICc	ΔAICc	Model weight
**1**	November rain + Bank vole autumn density + Bank vole spring density	-38.32	88.28	0	0.2
2	Rainy days in November + Bank vole autumn density + Bank vole spring density	-39.08	89.8	1.52	0.09
3	November rain + Bank vole autumn density + Bank vole spring density + Winter temperature	-38.02	92.05	3.76	0.03
4	November rain + Bank vole autumn density + Bank vole spring density + Rainy days in winter	-38.05	92.11	3.82	0.03
5	November rain + Bank vole autumn density + Bank vole spring density + Winter rain	-38.17	92.34	4.06	0.03

All candidate variables: November rain (mm), Rainy days in November, November T (C°, mean), October rain (mm), Rainy days in October, Rainy days in Autumn (= October and November combined as one variable), Bank vole autumn density, Bank vole spring density, Rainy days in winter, Winter rain (mm), Winter T (C°, mean)
